# Brain dynamics: the temporal variability of connectivity, and differences in schizophrenia and ADHD

**DOI:** 10.1038/s41398-021-01197-x

**Published:** 2021-01-21

**Authors:** Edmund T. Rolls, Wei Cheng, Jianfeng Feng

**Affiliations:** 1 Institute of Science and Technology for Brain-Inspired Intelligence, Fudan University, 200433 Shanghai, PR China; 2 Department of Computer Science, University of Warwick, Coventry, CV4 7AL UK; 3 Oxford Centre for Computational Neuroscience, Oxford, UK

**Keywords:** ADHD, Schizophrenia

## Abstract

We describe advances in the understanding of brain dynamics that are important for understanding the operation of the cerebral cortex in health and disease. In data from 1017 participants from the Human Connectome Project, we show that early visual and connected areas have low temporal variability of their functional connectivity. We show that a low temporal variability of the connectivity of cortical areas is related to high mean functional connectivity between those areas, and provide an account of how these dynamics arise. We then investigate how these concepts help to understand brain dynamics in mental disorders. We find that in both first episode and long-term schizophrenia, reduced functional connectivity of early visual and related temporal cortex areas is associated with increased temporal variability of the functional connectivity, consistent with decreased stability of attractor networks related to sensory processing. In ADHD, we find these functional connectivities are increased and their temporal variability is decreased, and relate this to increased engagement with visual sensory input as manifest in high screen time usage in ADHD. We further show that these differences in the dynamics of the cortex in schizophrenia, and ADHD can be related to differences in the functional connectivity of the specific sensory vs. association thalamic nuclei. These discoveries help to advance our understanding of cortical operation in health, and in some mental disorders.

## Introduction

Resting-state functional magnetic resonance imaging (rs-fMRI) techniques have contributed significantly to our understanding of brain activity, both in health and disease^1–4^. Classical methods are based on the continuous fluctuation of the brain blood oxygen level dependent (BOLD) signal. Functional connectivities (FC) then can be measured between spatially separated brain regions, in terms of correlation, coherence, and spatial grouping based on temporal similarities^5^.

In addition, the dynamical fluctuations of the BOLD signals, and how they relate to interactions between brain areas, is a topic of developing interest^6,7^. Here, we focus on the temporal variability of the functional connectivity between brain regions. We investigate whether the temporal variability of functional connectivity between a pair of brain regions is understandable and predictable from other measures, such as the mean functional connectivity between those brain regions.

When we made progress with addressing these aims in a large-scale investigation using data from 1017 participants from the Human Connectome Project (HCP), we wished to further test the hypotheses, and assess their utility, by examining data from participants with mental disorders, who might have differences in the measures investigated, and which might therefore help to support the answers provided by investigation of the healthy volunteers in the HCP dataset. We also hoped that investigation in these participants with the mental disorders schizophrenia and ADHD might help to elucidate how differences in brain dynamics may be related to these disorders.

The temporal variability of functional connectivity measures how the functional connectivity of a brain region with other brain regions alters across different time windows, which may reflect the dynamical reconfiguration of a brain region into different functional modules at different times^8,9^. Differences in the temporal variability of functional connectivity have been found to be related to creativity^9^ and mental disorders^8^.

This research goes beyond previous research by (1) relating temporal variability to the underlying mean functional connectivity between pairs of brain regions; (2) showing how these processes are different in both chronic and first-episode schizophrenia, and in ADHD; (3) relating these differences to the concepts of noise vs. stability in these mental disorders; and (4) showing for the first time how differences in the functional connectivity of different thalamic nuclei are closely related to the differences in brain dynamics in schizophrenia and ADHD, thus producing a substantial advance in our understanding of how the functioning of different parts of the thalamus is related to schizophrenia and ADHD.

## Materials and methods

### Participants

Four large-scale datasets were used in this investigation.

#### Human Connectome Project dataset

The dataset was selected from the Mar 2017 public data release from the Human Connectome Project (HCP, *n* = 1200), WU-Minn Consortium. Our sample includes 1017 subjects (ages 22–35 years, 546 females) scanned on a 3-T Siemens connectome-Skyra scanner, each with four resting state scans. Further details of the subjects, and the collection and preprocessing of the data are provided in the Supplementary Material, at the HCP website (http://www.humanconnectome.org/), and in a previous study^10^.

#### Chronic schizophrenia dataset

123 patients and 136 matched healthy controls were recruited from the Veteran General Hospital in Taipei, Taiwan. All participants were diagnosed according to the Diagnostic and Statistical Manual of Mental Disorder-IV criteria for schizophrenia, and each participant’s history of medical disease, psychiatric illness, and medication use was evaluated by interview and medical charts, with details in the Supplementary Material and in a previous study^11^.

#### First episode schizophrenia dataset

The dataset contained 266 subjects (154 patients and 112 healthy controls) recruited from the Shanghai Mental Health Center. The first episode schizophrenia patients were identified according to DSM-IV criteria for schizophrenia by qualified psychiatrists^12^, and the Positive and Negative Syndrome Scale (PANSS)^13,14^ was used to assess symptom severity, with details in the Supplementary Material and in a previous study^15^.

#### ADHD dataset

The fMRI data used were from the ADHD-200 Consortium (https://fcon_1000.projects.nitrc.org/indi/adhd200/) from the Institute of Mental Health and National Key Laboratory of Cognitive Neuroscience and Learning (Peking University, Beijing, China). This dataset included 239 children, with 142 healthy controls, and 97 participants with ADHD. Details of the subjects and the collection of the data are provided in the Supplementary Material and in a previous study^16^.

### **T**he whole-brain functional connectivity network

Preprocessing was performed using the HCP pipeline, as described in detail in the Supplementary Material. Care was taken to ensure that the head motion parameters were regressed out and structured artifacts were removed by ICA + FIX processing (Independent Component Analysis followed by FMRIB’s ICA-based X-noiseifier^17,18^) (see Supplementary Material). The automated anatomical labeling atlas 3 (AAL3) template^19^ was used to construct the whole-brain functional connectivity network. This atlas was used because it defines well not only the orbitofrontal cortex, but also the thalamic nuclei^19^, and also because it has proved useful in understanding the functional^20^ and anatomical^21^ connectivity between different brain areas. The different AAL3 areas are shown in Table S1. This atlas has the advantages that it has named brain areas that can be related to neurology, and because the divisions it makes for a number of brain areas including the orbitofrontal and cingulate cortices reflect also the parcellation that is found based on functional connectivity^20^. The 132 brain region time series (BOLD signals, and excluding the cerebellum and regions with less than five voxels (i.e., Thal_Re, VTA, LC and Raphe) were extracted by averaging voxel time series within each AAL3 region. Then, for each subject, the Pearson cross-correlations between all pairs of regional BOLD signals were calculated to reflect the functional connectivity between region pairs. A brain functional connectivity network that consisted of the 132 brain regions and 8646 functional connectivity links between them was constructed.

### Temporal variability of the functional connectivity profile of a brain region

The temporal variability of a brain region was obtained by correlating the FC profile of a brain region across different time windows, which reflects the dynamical reconfiguration of a brain region into distinct functional modules at different times^8^. To characterize the temporal variability of the functional connectivity of a given AAL3 brain region, we first segmented all BOLD signals into *n* non-overlapping windows with length *l*. The whole-brain FC network *F*_i_ (an *m* * *m* matrix, with *m* = 132 nodes) in the *i*th time window was then constructed, with the Pearson correlation being the measure of FC. The FC profile of region *k* at time window *i* is denoted by *F*_i_ (*k*:) (shortened as *F*_i,k_), which is an *m*-dimensional vector that represents all the functional connections of region *k*. The variability of a region *k* is defined as:

$$V_{\rm{k}} = 1 - \overline {{\rm{corrcoef}}\left( {F_{{\rm{i,k}}}, F_{{\rm{j,k}}}} \right)} \;i,j = 1,2,3, \ldots ,n,\;i \,\ne\, j$$

We calculated *V*_k_ at a number of different window lengths (*l* = equal to 20, 22, 24,…40 s) and then took the average value as the final variability to avoid arbitrary choice of window length.

## Results

### **T**he temporal variability of the functional connectivity of brain regions is related to the mean functional connectivity between regions

**Fig. 1:**
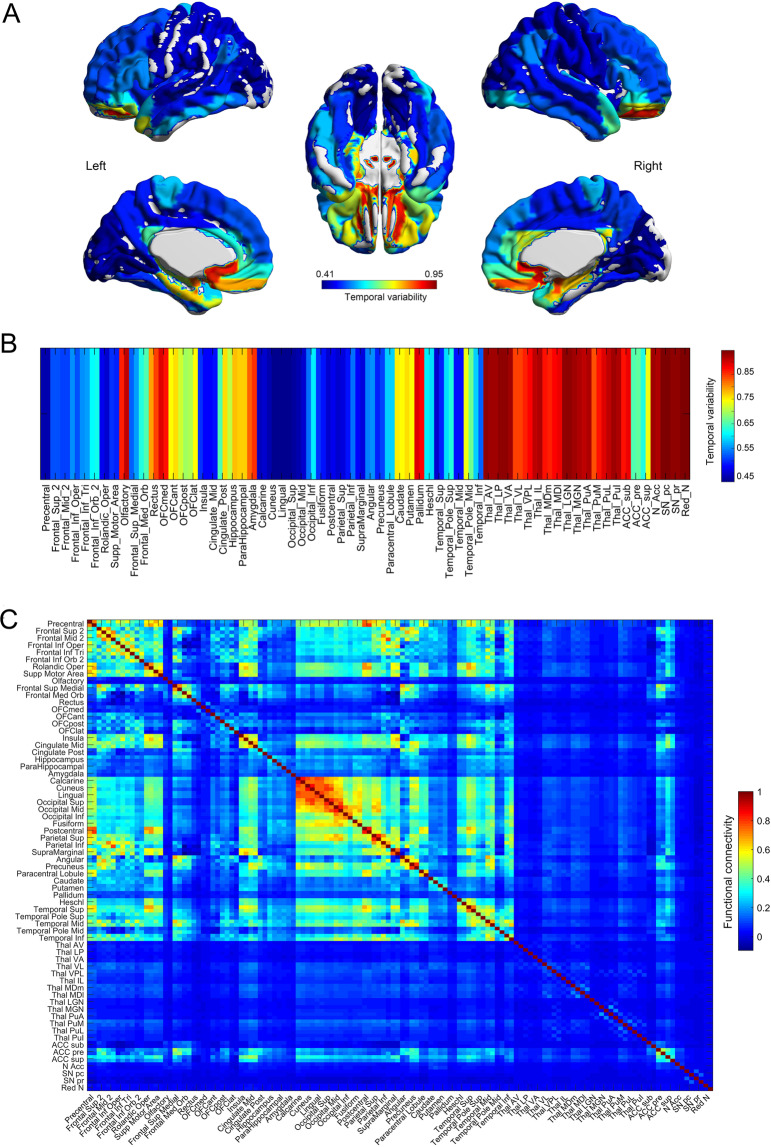
The temporal variability of functional connectivity is related to the mean functional connectivity of brain regions. **A** A brain surface diagram (based on AAL3) showing the temporal variability of the functional connectivity of different brain regions. The data are from the Human Connectome Project (HCP). **B** The temporal variability of the functional connectivity of different AAL3 regions. **C** The functional connectivity between AAL3 areas from the HCP.

We show in Fig. 1B (right) the temporal variability of the functional connectivity of different AAL3 regions for the 1017 HCP participants. Figure 1A provides a brain surface diagram showing the temporal variability of the functional connectivity of different brain regions. Figure 1A shows that areas with high temporal variability of the functional connectivity include the amygdala, orbitofrontal cortex, caudate nucleus, cingulate cortex, hippocampus and parahippocampal gyrus, and the Lateral Geniculate and Medial Geniculate nuclei of the thalamus. Areas with low temporal variability of the functional connectivity include early visual cortical areas (lingual, occipital, and calcarine, and also the fusiform gyrus); and motor areas (precentral and postcentral, and Rolandic cortex).

We can develop an understanding of the relationship between the temporal variability of the functional connectivity of different brain regions in terms of the underlying mean resting state functional connectivity for the 1017 HCP participants, illustrated in Fig. 1C. This leads to the following hypotheses. Early visual cortical areas have low temporal variability, and high functional connectivity between them. The high functional connectivity between them makes them all tend to move together dynamically, because of their strong connections with each other, keeping the temporal variability measure low. This contrasts with other areas such as the hippocampus and parahippocampal gyrus, amygdala, and orbitofrontal cortex that have high temporal variability of the functional connectivity, relating it is suggested to their connections with many brain regions (like hubs in a graph), and can all therefore move independently of each other dynamically, because they have connections with many brain areas. For early visual cortical areas in the above, we can also include conceptually some other areas that are strongly connected with them, but not with widespread areas of the brain. These other areas include some motor areas such as premotor cortex, precentral, and postcentral cortex, supplementary motor area, and perhaps the basal ganglia (putamen and pallidum especially).

Thus a key to understanding temporal variability of the functional connectivity is the mean functional connectivity, we propose, in the ways just outlined.

### Higher temporal variability of the functional connectivity for some brain regions in schizophrenia is associated with lower mean functional connectivity

#### Chronic schizophrenia

**Fig. 2:**
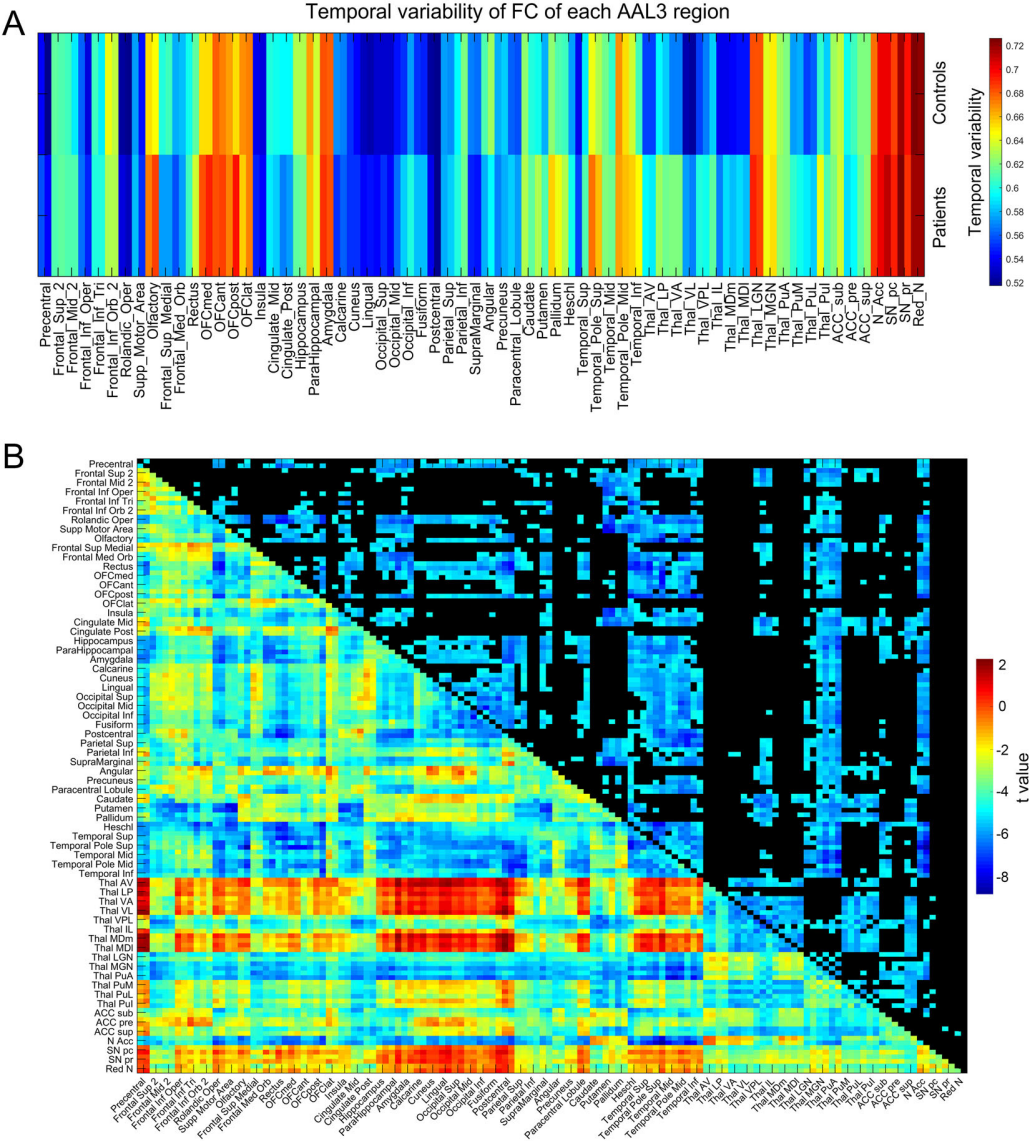
Higher temporal variability of functional connectivity of some brain regions in chronic schizophrenia. **A** The temporal variability of the functional connectivity of different AAL3 regions in the chronic schizophrenic and control groups. **B** The functional connectivity of AAL3 areas for the chronic schizophrenic group minus controls. The lower left shows the *t*-value for the difference in functional connectivity of patients - controls; the upper right shows the significantly different functional connectivities after Bonferroni correction.

We next investigated how temporal variability of the functional connectivity may be different in chronic schizophrenia, using data from 123 patients and 136 matched healthy controls (see “Methods” section). Figure 2A shows that areas with significantly higher temporal variability of the functional connectivity in chronic schizophrenia included some early visual cortical areas (inferior occipital, fusiform) and the temporal lobe areas to which they are connected (e.g., TemporalInf and TemporalPoleSup); the orbitofrontal cortex (and OLF which is closely connected with it); association thalamic nuclei including MD (which connects with the orbitofrontal cortex), VA and VL; and some motor areas including the putamen and pallidum (see also Table S2).

Consistent with the analysis described above, the functional connectivities involving many of the AAL3 regions with a higher temporal variability of the functional connectivity are lower in chronic schizophrenia (Fig. 2B), including the early visual (occipital, calcarine, lingual, and cuneus) cortical areas between themselves, and between them and the temporal lobe areas to which they are connected (typical *t*-values −6.5, *p* < 10^−7^), some motor areas (e.g., precentral gyrus and Rolandic operculum, typical *t*-values −6, *p* < 10^−6^), and posterior orbitofrontal cortex (*t* = −5.34, *p* = 2 × 10^−7^). To provide more detail: the mean functional connectivity within early visual cortical areas (occipital, calcarine, lingual, and cuneus) was 0.78 in the individuals with chronic schizophrenia, compared to 0.85 in the controls, *t* = −5.93; *p* = 1 × 10^−8^. Early auditory cortical areas, in particular Heschl’s gyrus, and also the superior temporal gyrus which includes auditory areas, also had significantly lower functional connectivity in the group with chronic schizophrenia (Fig. 2B). Figure 2B also shows for the first time that different thalamic nuclei are differently affected in chronic schizophrenia: the association thalamic nuclei, including MD, VA, VL and VP have higher functional connectivity in chronic schizophrenia; and the specific sensory nuclei LGN (visual) and MGN (auditory) have lower functional connectivity in schizophrenia. The implications of these interesting differences are considered in the Discussion.

#### First episode schizophrenia

We start by summarizing that the findings in first episode schizophrenia (with 154 patients and 112 healthy controls) are overall similar to those found in the chronic group, but smaller in magnitude.

**Fig. 3:**
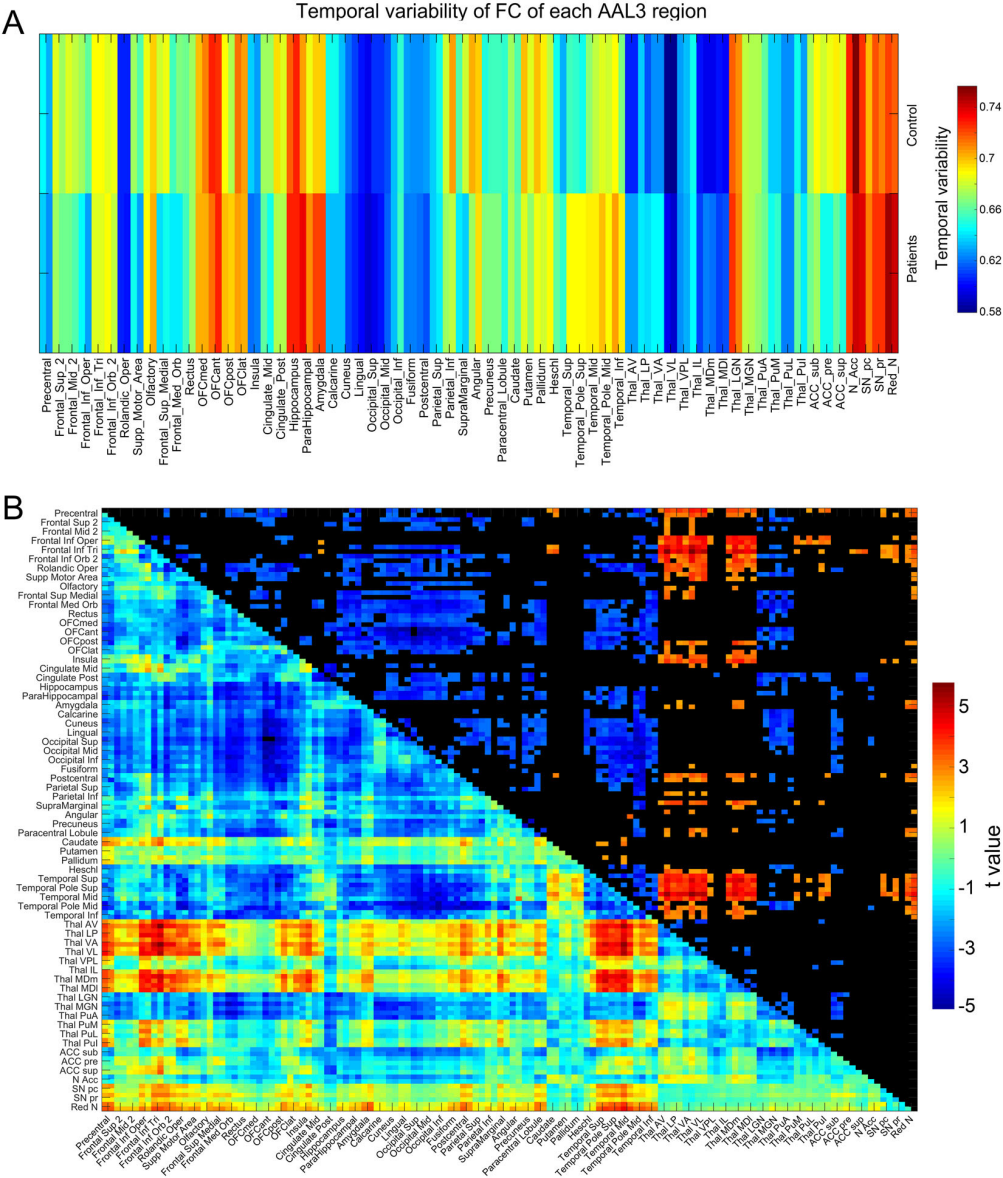
Higher temporal variability of functional connectivity of some brain regions in first episode schizophrenia. **A** The temporal variability of the functional connectivity of different AAL3 regions in the first episode schizophrenic and control groups. **B** The functional connectivity of AAL3 areas for the first episode schizophrenic group minus controls. The lower left shows the *t*-value for the difference in functional connectivity of patients - controls; the upper right shows the significantly different functional connectivities after FDR correction, with red showing higher and blue showing lower functional connectivity in patients - controls.

Figure 3A shows that areas with higher temporal variability of the functional connectivity in first episode schizophrenia included the temporal cortex connected to early visual cortical areas; anterior cingulate cortex; and superior medial prefrontal cortex (with statistics provided in Table S3). In first episode schizophrenia, there was no marked difference in the temporal variability of the functional connectivity of early visual cortical areas.

Figure 3B shows that, consistent with what is shown in Fig. 2B, the temporal lobe functional connectivities are lower in first episode schizophrenia; and so are the orbitofrontal cortex and hippocampus and parahippocampal gyrus functional connectivities especially with the temporal lobe and early visual cortical areas. In more detail, the functional connectivities of early visual cortical areas with temporal lobe cortical areas were lower in first episode schizophrenia (typical *t*-values 3.0–4.5, typical *p* values 10^−3^ to 10^−5^); and with the orbitofrontal cortex (typical *t* values 3.0–4.5, typical *p* values 10^−3^ to 10^−5^); and with the hippocampus and parahippocampal gyrus (typical *t* values 3.4–3.7, typical *p* values 10^−3^). The finding that there was lower functional connectivity between the early visual cortical areas was similar to what was found in chronic schizophrenia, but the difference was smaller in first episode schizophrenia and not quite significant (*t* = −1.81, *p* = 0.07). FrontalInfTri and FrontalInfOperc (BA 45 and 44, Broca’s area on the left) have significantly reduced functional connectivity, especially with the hippocampus, early visual cortical areas, and the temporal cortical areas. Figure 3B also shows that in first episode schizophrenia the association thalamic nuclei, including MD, VA, VL, LP, and VP have higher functional connectivity; and the specific sensory nuclei LGN and MGN have lower functional connectivity in first episode schizophrenia (for example for connectivity with early visual cortical areas, typical *t* values are 2.5–3, with typical *p* values 0.01–0.001). This is the same pattern as in the chronic schizophrenia dataset illustrated in Fig. 2B.

### **L**ower temporal variability of the functional connectivity for some brain regions in ADHD is associated with higher functional connectivity

**Fig. 4:**
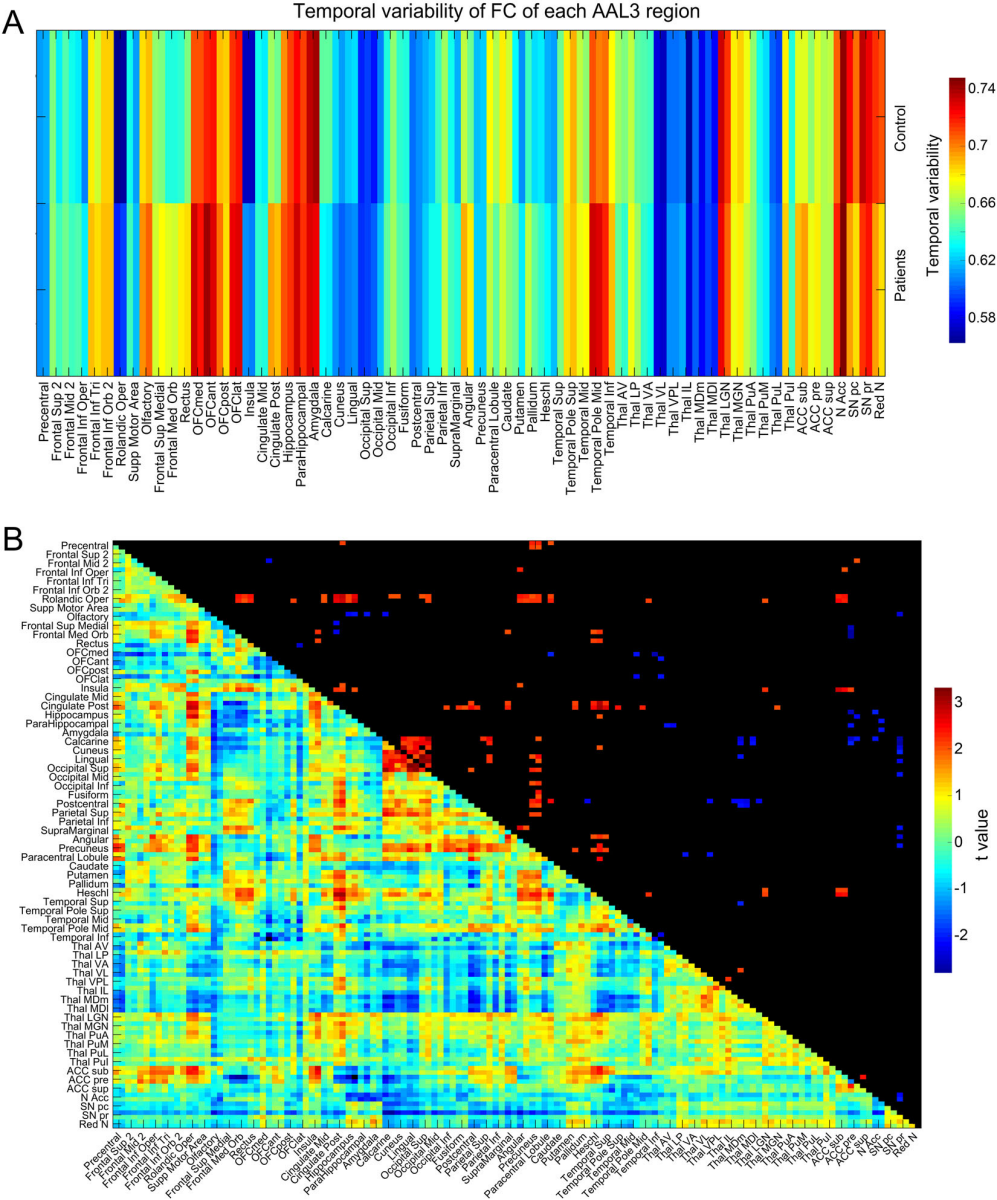
Lower temporal variability of functional connectivity of some brain regions in ADHD. **A** The temporal variability of the functional connectivity of different AAL3 regions in the ADHD and control groups. **B** The functional connectivity of AAL3 areas for the ADHD group minus controls. The lower left shows the *t*-value for the difference in functional connectivity of patients - controls; the upper right shows the significantly different functional connectivities with *p*<0.05, with red showing higher and blue showing lower functional connectivity in patients - controls.

In a group of 94 children with ADHD and 142 healthy controls (see “Methods” section), Fig. 4A shows that areas with lower temporal variability of the functional connectivity in ADHD include early visual cortical areas (cuneus). Figure 4A also shows that areas with higher temporal variability of the functional connectivity in ADHD included the gyrus rectus, VMPFC (FrontalMedOrb), and posterior cingulate cortex. Statistics are provided in Table S4.

In relation to the decreases of the temporal variability of the functional connectivity in ADHD, it is consistent, in the light of the hypotheses generated here, that there is higher mean functional connectivity in ADHD of early visual areas with each other; and with the precuneus (*t* = 2.3 *p* = 0.02); superior parietal cortex (*t* = 2.23 *p* = 0.02); and a tendency for higher functional connectivity with the LGN and MGN, and lower functional connectivity with the associative thalamic nuclei such as MD, VA, and VL (Fig. 4B). In more detail: the mean functional connectivity within early visual cortical areas (in particular calcarine, cuneus, lingual and superior occipital) was 0.87 in the people with ADHD compared to 0.836 in the controls, *t* = 3.0; *p* = 0.003. Early auditory cortical areas, in particular Heschl’s gyrus, also had higher functional connectivity in the group with ADHD (Fig. 4B).

## Discussion

We found in four replications with different datasets (Figs. 1–4) that the temporal variability of the functional connectivity of different AAL3 brain regions is low in areas such as the early visual cortical areas with high mean (i.e., averaged over the whole of the resting-state fMRI session) functional connectivity with each other (Fig. 1C); and is high in areas such as the orbitofrontal cortex, amygdala, and hippocampus which have relatively low functional connectivity with early visual cortical areas, as well as connectivity with a number of different brain regions in a hub-like way (Fig. 1C). We propose the because of the high positive interconnectivity of the early visual cortical areas, they tend to act together to support activity in each other, and therefore have relatively low temporal variability of their functional connectivity.

This provides an account of temporal variability of the functional connectivity in terms of the underlying mean functional connectivity (Fig. 1). These hypotheses are supported by the finding of lower functional connectivity of early visual cortical areas, and increased temporal variability of the functional connectivity of these areas, in schizophrenia (Figs. 2 and 3); and higher functional connectivity of early visual cortical areas and decreased temporal variability of the functional connectivity of these areas in ADHD (Fig. 4).

Quantitative approaches to attractor neuronal networks of the type found in the cerebral cortex provide a firm foundation for understanding the relation between the functional connectivity that couples neurons within or between attractor networks, and the stability vs. temporal variability of the functional connectivity^22–25^, including in the context of schizophrenia^26–28^. If the excitatory connections within or between the neuronal populations are high, then this promotes deep basins of attraction in which the energy is low^29^. Once the system has entered such a basin of attraction, the noise in the system contributed by the stochastic spiking times of the neurons is rarely sufficiently high for the system to escape from the basin of attraction, so the neurons are not changing their firing rates as the system moves from one basin of attraction to another. If the excitatory connections have a low value, then the depth of the basin of attraction is low, and the temporal variability of the firing is high as the system moves from one attractor state to another, as we have shown quantitatively in an integrate-and-fire attractor network model of the dynamics of schizophrenia^26–28^. The fluctuations that cause these transitions can be the randomness in the spike times of the neurons, as has been shown by simulating systems of different sizes^22–28,30,31^. These transitions from one coalition of neurons or networks being active in one attractor to another coalition can be facilitated by the typical adaptation in the firing rates of cortical neurons, as we have shown^32–34^. Moreover, when there is a transition to another set of neurons that form a coalition in another attractor or set of attractors, there may be a synchronized peak of neuronal activity in the new coalition because its neurons are de-adapted^24,32–34^. The measured functional connectivity may be higher between brain systems when they are in the same attractor, for then their activity becomes coherent, and this coherence may increase the signal to noise ratio of the measured functional connectivity^35^. Further, if a brain region has connections with a number of brain systems, any one of these subsystems may take over as the next coalition, and the brain region can be thought of as a hub. In the context of schizophrenia, it is proposed that reduced synaptic efficacy of brain regions such as the prefrontal cortex areas, due for example to spine loss^36^, contributes to greater temporal variability of the functional connectivity and instability of cortical attractor networks, and therefore to difficulty in maintaining attention without mind-wandering that involves too much associativity with loosely related thoughts^26,28^. In the present context, an implication is that the reduced mean functional connectivity of early visual and related temporal cortex areas is associated with increased temporal variability of the neuronal firing and the measured functional connectivity as new coalitions of neurons or attractors become active, consistent with decreased stability of attractor networks related to sensory processing. It is further proposed that this reduced mean functional connectivity shifts the balance of processing in schizophrenia away from early sensory processing, and instead towards thoughts that are more internal and less influenced by inputs from the external world. The advantage of considering temporal variability of the functional connectivity rather than only mean functional connectivity is that temporal variability of the functional connectivity involves the dynamics of the brain. This provides a clear bridge to understanding how the dynamical processing inside the brain is related to the symptoms of schizophrenia, including the reduced stability of attention, and the tendency to jump to only loosely associated thoughts, because temporal variability of the functional connectivity/stochasticity is high^22–28^. Another advantage is that cortical processing can be considered as involving noisy transitions from one state to another, with the extent of these transitions, and how all cortical areas interact, influenced by the depths of the local basins of attraction and whether they are in an adapted state because of recent activity^23,25,37,38^.

In relation to chronic schizophrenia, it was found that areas with higher temporal variability of the functional connectivity included some early visual cortical areas (inferior occipital and fusiform) and some temporal cortex areas connected to these motor areas (e.g., pallidum), some medial orbitofrontal cortex areas (OFCmed and OFCpost), and the “associative” thalamic nuclei MD, VA, and LP (Fig. 2A). These higher temporal variabilities in schizophrenia were associated with lower mean (i.e., across time) functional connectivity of these brain regions (Fig. 2B), and this is consistent with the hypothesis that a factor in schizophrenia is a reduction in the connectivity and therefore excitability of some brain regions, which destabilizes attractor networks in these regions because the firing rates are insufficient to maintain the networks in a high firing rate state^11,22,26^.

In first episode schizophrenia the differences in the temporal variability of functional connectivity and in the functional connectivity (Fig. 3A, B) were qualitatively similar to those in chronic schizophrenia, though smaller, and as these were independent datasets, this provides a cross-validation of the findings. The finding that there was no significant difference in early visual cortical areas of functional connectivity between themselves (Fig. 3B) is a difference from chronic schizophrenia, although there was lower functional connectivity of early visual cortical areas with some temporal lobe areas in first episode schizophrenia, with the corresponding increase in the temporal variability of this functional connectivity.

The weak interconnectivity of early visual cortical areas is consistent with the hypothesis that individuals with chronic schizophrenia are less influenced by “bottom-up, sensory” visual stimuli in the environment, and instead their cognitive state is relatively detached from the world, so that their thoughts are more dominated by internal processing. Consistent with that, the “top-down, backward” effective connectivity in schizophrenia is relatively unchanged, but the “bottom-up, forward” effective connectivity is lower in people with schizophrenia^11^, and this would also tend to promote a tendency to stay locked in internal thoughts somewhat detached from the real world.

The finding in ADHD of higher functional connectivity of early visual cortical areas with each other and decreased temporal variability of the functional connectivity of these areas (Fig. 4) may be relevant to understanding ADHD better. It is consistent with the hypothesis that high mean functional connectivity between these early visual areas helps to lock them together, and thus to reduce their temporal variability of the functional connectivity. It is of interest that both these effects in ADHD are in the reverse direction to what is found in chronic schizophrenia (shown in Fig. 2). The strong interconnectivity of early visual cortical areas is consistent with the hypothesis that individuals with ADHD are strongly influenced by visual stimuli in the environment, and become locked into these, rather than finding it easy to pay attention to non-sensory tasks such as continuous performance tasks, and anything that will be impaired by strong visual sensory input. Consistent with this neurobiological approach to ADHD, adolescents with a high screen use (TV, mobile phone, computers, and computer games) tend to have a high ADRS (attention-deficit risk score)^39^.

Figures 2B and 3B show that in chronic and first episode schizophrenia, the association thalamic nuclei, including MD, VA, VL, and VP have higher functional connectivity; and the specific sensory nuclei LGN and MGN (and the associated anterior pulvinar) have lower functional connectivity. The lower functional connectivity of these specific sensory thalamic nuclei may relate to the lower mean (across time) functional connectivity of some visual cortical areas, some of which have higher temporal variability (Figs. 2 and 3). The higher functional connectivity of the association thalamic nuclei (MD etc) in schizophrenia is with orbitofrontal and temporal lobe areas (Fig. 2B), which tend to have higher temporal variability of the functional connectivity in schizophrenia, and this may account for the higher temporal variability of the functional connectivity of these association thalamic nuclei. The important point here is that the thalamus should no longer be regarded as a unit in schizophrenia: the association thalamic nuclei have *higher* mean functional connectivity in schizophrenia, and the specific sensory LGN, MGN and PuA nuclei have *lower* mean functional connectivity in schizophrenia. Moreover, we propose that the latter is important for understanding schizophrenia, and is related to some insensitivity to especially visual sensory inputs from the world. Very interestingly, there is a strong dopamine input to most of the association thalamic nuclei in primates, and much less to the lateral and medial geniculate nuclei^40^. There is also higher functional connectivity of the substantia nigra with the association thalamic nuclei in schizophrenia than controls (Figs. 2B and 3B, and not in ADHD Fig. 4B). This suggests that an important way in which dopamine may be involved in schizophrenia^26^ is by its differentially greater effects on the association thalamic nuclei relative to the lateral and medial geniculate nuclei. We propose that in schizophrenia this biases processing away from external visual and auditory inputs, and towards internal cognitive processing in associative cortical areas such as the prefrontal and temporal cortical areas.

An earlier investigation reported that mean functional connectivity differences involving the thalamus were prominent in chronic rather than in first episode schizophrenia^41^. The present investigation considerably extends that, by showing that the specific thalamic nuclei such as the lateral and medial geniculate have reduced mean functional connectivity in schizophrenia, that the association thalamic nuclei (such as MD, VA, and VL) have increased mean functional connectivity in schizophrenia, and by showing that these are present in first episode as well as chronic schizophrenia, though are larger in chronic schizophrenia. Highlights of these new results are that they were obtained with a new and different population of patients with first-episode schizophrenia, and with the automated anatomical labeling atlas 3 (AAL3) which includes a parcellation of the thalamic nuclei^19^.

Consistent with these hypotheses about the thalamus, the mean (across time) functional connectivity of the visual sensory thalamic nuclei is higher in people with ADHD than in controls, and this is related to their increased sensitivity to visual sensory input; and the mean functional connectivity of the association thalamic nuclei (MD, VA, etc) is lower in people with ADHD, which is associated with more variability of functional connectivity in brain areas such as the ventromedial prefrontal cortex (FrontalMedOrb), and orbitofrontal cortex, and other frontal areas that may be related to the inability to maintain attention and executive function, in which these cortical areas are involved^22^.

## Conclusions

The research described here leads to the following conclusions:

1. Early visual and connected areas have low temporal variability of the functional connectivity.

2. We show that a low temporal variability of the functional connectivity of cortical areas is related to high mean functional connectivity between those areas, and provide an account of how these dynamics arise.

The analysis of these brain dynamics and their origin was supported by differences found in some mental disorders, which enhance our understanding of these disorders, as follows.

3. In both first episode and long-term schizophrenia, reduced functional connectivity of early visual and related temporal cortex areas is associated with increased temporal variability of the functional connectivity, consistent with decreased stability of attractor networks related to sensory processing or the external world.

4. In ADHD these mean functional connectivities are increased and their temporal variability is decreased. We relate this to increased engagement with visual sensory input as manifest in high screen time usage in ADHD.

5. Differences in the dynamics of the cortex in schizophrenia and ADHD can be related to differences in the functional connectivity of the specific sensory vs. association thalamic nuclei.

These discoveries were made possible by the analysis of four separate large samples of neuroimaging data, were facilitated by use of the AAL3 atlas which includes parcellation of the thalamus, and help to advance our understanding of cortical operation in health, and in some mental disorders.

## Supplementary information

Supplemental Material

## Data Availability

The dataset for the Human Connectome project is available at http://www.humanconnectome.org/). The ADHD dataset is available from the ADHD-200 Consortium website https://fcon_1000.projects.nitrc.org/indi/adhd200/. Details of the other clinical data are available from the corresponding author. Standard code functions available in Matlab and SPM were used.

## Code availability

Standard code functions available in Matlab and SPM were used.
