# Supplementary material for: Brain dynamics: the temporal variability of connectivity, and differences in schizophrenia and ADHD
Source: Transl Psychiatry. 2021 Jan 21;11:70. doi: 10.1038/s41398-021-01197-x (PMC7820440; doi:10.1038/s41398-021-01197-x)
Supplement: Supplementary file 1 — Supplemental Material [file 41398_2021_1197_MOESM1_ESM.docx]

**Brain dynamics: temporal variability of connectivity,**

**and differences in schizophrenia and ADHD**

**Supplementary Material**

**Translational Psychiatry (2021)**

Edmund T Rolls^1,2,3^, Wei Cheng^1^ and Jianfeng Feng^1,2^

1. Institute of Science and Technology for Brain-inspired Intelligence, Fudan University, Shanghai, 200433, PR China

2. Department of Computer Science, University of Warwick, Coventry CV4 7AL, UK

3. Oxford Centre for Computational Neuroscience, Oxford, UK

**Participants**

*Human Connectome Project dataset*

The dataset was selected from the Mar 2017 public data release from the Human Connectome Project (HCP, N = 1200), WU-Minn Consortium. The sample included 1017 subjects (ages 22–35 years, 546 females) scanned on a 3-T Siemens connectome-Skyra scanner. Two resting state fMRI acquisitions on different days were used. The four resting-state runs of approximately 15 minutes each were acquired in separate sessions on two different days, with the eyes open with relaxed fixation on a projected bright cross-hair on a dark background. The WU-Minn HCP Consortium obtained full informed consent from all participants, and research procedures and ethical guidelines were followed in accordance with the Institutional Review Boards (IRB). The major parameters for the neuroimaging acquisition were as follows ^1^. Resting-state fMRI data were acquired using a 3T MRI scanner (Siemens) in a 15-min period in which the participants were awake in the scanner. A total of 1200 volumes of images were obtained (TR/TE: 720/33 ms, Flip angle 52 degree, matrix size: 104×90, FOV = 208×180 mm^2^, slices 72). Further details of the subjects, and the collection and preprocessing of the data are provided at the HCP website (<http://www.humanconnectome.org/)> and in previous studies ^2^.

*Chronic schizophrenia dataset*

123 patients and 136 matched healthy controls were recruited from the Veteran General Hospital in Taipei, Taiwan. All participants were diagnosed according to the Diagnostic and Statistical Manual of Mental Disorder-IV criteria for schizophrenia, and each participant's history of medical disease, psychiatric illness, and medication use was evaluated by interview and medical charts carefully. Experiments were conducted in accordance with the Declaration of Helsinki and approved by the Institutional Review Board of Taipei Veterans General Hospital. Written informed consent was obtained from all participants after ensuring adequate understanding of the study. Any participants with the following conditions were excluded: (1) a comorbid substance-related disorder, (2) presence of neurobiological disorders, such as dementia, head injury, stroke, or Parkinson’s disease; (3) presence of hypertension, diabetes, hyperlipidemia or coronary heart disease; (4) severe medical illness, such as malignancy, heart failure, or renal failure; (4) presence of ferromagnetic foreign bodies or implants that were anywhere in the body. Clinical severity was evaluated by the psychiatrist-assessed PANSS score.

The major parameters for the neuroimaging acquisition were as follows. Resting-state fMRI data were acquired using a 3T MRI scanner (Siemens) in an 8-min period in which the participants were awake in the scanner. A total of 200 volumes of images were obtained (TR/TE: 2500/27 ms, Flip angle 77 degree, matrix size: 64×64, voxel size: 3.44×3.44×3.4 mm^3^; FOV = 220×220 mm2, slices 50). The eyes were closed during the resting state fMRI. More details of the subjects and the collection of the data were described in a previous study ^3^.

*First episode schizophrenia dataset*

The dataset used for this investigation contains 266 subjects (154 patients and 112 healthy controls) recruited from the Shanghai Mental Health Center. All the subjects were Mandarin-speaking Han Chinese individuals from the Shanghai metropolitan area. The first episode schizophrenia patients were identified according to DSM-IV criteria for schizophrenia by qualified psychiatrists ^4^, and the Positive and Negative Syndrome Scale (PANSS) ^5^ was used to assess symptom severity. Using DSM-IV criteria, the healthy controls were all confirmed to be free of schizophrenia or other Axis 1 disorders and without a history of substance abuse or clinically significant head trauma. All participants were between the ages of 14 and 45 years; were right-handed; had no history of substance abuse or suicidal ideation; and had no MRI contraindications. The major parameters for neuroimaging collection were as follows. Resting-state fMRI data were acquired using a 3T MRI scanner (Siemens) in an 8-min period in which the participants were awake in the scanner. A total of 240 volumes of images were obtained (TR/TE: 2000/30 ms, Flip angle 77 degree, matrix size: 64×64, voxel size: 3 × 3 × 3 mm3; FOV = 220×220 mm2, slices 50). The eyes were closed during the resting state fMRI. More details of the subjects and the collection of the data are described in a previous study ^6^.

*ADHD dataset*

The fMRI data used in this paper are from the ADHD-200 Consortium for the global competition (http://fcon_1000.projects.nitrc.org/indi/adhd200/). Since the fMRI data collected from different centers may have some systematic differences that are possibly caused by the fMRI machine used, in this paper we only use the fMRI data collected from the Institute of Mental Health and National Key Laboratory of Cognitive Neuroscience and Learning (Peking University, Beijing, China) to minimize variability across institutions. This dataset included 239 children, 142 of which are healthy controls (59 females, 83 males; mean age 11.45 ± 1.84 years), and the rest 97 are patients with ADHD (11 females, 86 males; mean age 12.09 ± 2.05 years). All participants (ADHD and controls) were evaluated by the Schedule of Affective Disorders and Schizophrenia for Children—Present and Lifetime Version (KSADS-PL) with one parent for the establishment of the diagnosis. The ADHD Rating Scale (ADHD-RS) IV was employed to provide dimensional measures of ADHD symptoms ^7^. Additional inclusion criteria included: (1) right-handedness, (2) no lifetime history of head trauma with loss of consciousness, (3) no history of neurological disease and no diagnosis of schizophrenia, affective disorder, pervasive development disorder, and substance abuse, and (4) a full scale (WISCC-R) score greater than 80. The major parameters of neuroimaging collection were as follows. Resting-state fMRI data were acquired using a 3T MRI scanner (Siemens) in an 8-min period in which the participants were awake in the scanner. A total of 240 volumes of images were obtained (TR/TE: 2000/30 ms, Flip angle 90 degree, matrix size: 64×64, voxel size: 3.1×3.1×3.5 mm^3^; FOV = 220×220 mm^2^, slices 33). The procedure allowed the eyes to be either closed or open during the resting state fMRI. More details of the subjects and the collection of the data are provided in a previous study ^8^.

**Data preprocessing**

The HCP data pre-processing was carried out using FSL (FMRIB Software Library), FreeSurfer, and the Connectome Workbench software. All the data preprocessing procedures were performed by the Human Connectome Project (HCP) as described in^1^. The data preprocessing included correction for spatial and gradient distortions and head motion, intensity normalization and bias field removal, registration to the T1 weighted structural image, transformation to 2 mm Montreal Neurological Institute (MNI) space, and the FIX artefact removal procedure ^9, 10^. Finally, the head motion parameters were regressed out and structured artefacts were removed by ICA+FIX processing (Independent Component Analysis followed by FMRIB’s ICA-based X-noiseifier ^11, 12^. The data preprocessing pipeline developed by FMRIB (Oxford University Centre for Functional MRI of the Brain) used here has been widely used in resting state fMRI studies ^9, 13-15^. For the other datasets, the preprocessing used the same pipeline. This pipeline is efficient in removing noise, and regressing out the global signal etc is not involved ^11, 12^.

**Table S1a.** The anatomical regions defined in each hemisphere and their label in the automated anatomical labelling atlas AAL2 ^16^. The regions that have been redefined in AAL3 ^17^ are shown in italics. Column 4 provides a set of possible abbreviations for the anatomical descriptions.

| NO. | ANATOMICAL DESCRIPTION | LABEL  aal2.nii.gz | POSSIBLE  ABBREVIATION |
| --- | --- | --- | --- |
| 1,2 | Precentral gyrus | Precentral | PreCG |
| 3, 4 | Superior frontal gyrus, dorsolateral | Frontal_Sup | SFG |
| 5, 6 | Middle frontal gyrus | Frontal_Mid | MFG |
| 7, 8 | Inferior frontal gyrus, opercular part | Frontal_Inf_Oper | IFGoperc |
| 9, 10 | Inferior frontal gyrus, triangular part | Frontal_Inf_Tri | IFGtriang |
| 11, 12 | IFG pars orbitalis, | Frontal_Inf_Orb | IFGorb |
| 13, 14 | Rolandic operculum | Rolandic_Oper | ROL |
| 15, 16 | Supplementary motor area | Supp_Motor_Area | SMA |
| 17, 18 | Olfactory cortex | Olfactory | OLF |
| 19, 20 | Superior frontal gyrus, medial | Frontal_Sup_Med | SFGmedial |
| 21, 22 | Superior frontal gyrus, medial orbital | Frontal_Med_Orb | PFCventmed |
| 23, 24 | Gyrus rectus | Rectus | REC |
| 25, 26 | Medial orbital gyrus | OFCmed | OFCmed |
| 27, 28 | Anterior orbital gyrus | OFCant | OFCant |
| 29, 30 | Posterior orbital gyrus | OFCpost | OFCpost |
| 31, 32 | Lateral orbital gyrus | OFClat | OFClat |
| 33, 34 | Insula | Insula | INS |
| *35, 36* | *Anterior cingulate & paracingulate gyri* | Cingulate_Ant | ACC |
| 37, 38 | Middle cingulate & paracingulate gyri | Cingulate_Mid | MCC |
| 39, 40 | Posterior cingulate gyrus | Cingulate_Post | PCC |
| 41, 42 | Hippocampus | Hippocampus | HIP |
| 43, 44 | Parahippocampal gyrus | ParaHippocampal | PHG |
| 45, 46 | Amygdala | Amygdala | AMYG |
| 47, 48 | Calcarine fissure and surrounding cortex | Calcarine | CAL |
| 49, 50 | Cuneus | Cuneus | CUN |
| 51, 52 | Lingual gyrus | Lingual | LING |
| 53, 54 | Superior occipital gyrus | Occipital_Sup | SOG |
| 55, 56 | Middle occipital gyrus | Occipital_Mid | MOG |
| 57, 58 | Inferior occipital gyrus | Occipital_Inf | IOG |
| 59, 60 | Fusiform gyrus | Fusiform | FFG |
| 61, 62 | Postcentral gyrus | Postcentral | PoCG |
| 63, 64 | Superior parietal gyrus | Parietal_Sup | SPG |
| 65, 66 | Inferior parietal gyrus, excluding supramarginal and angular gyri | Parietal_Inf | IPG |
| 67, 68 | SupraMarginal gyrus | SupraMarginal | SMG |
| 69, 70 | Angular gyrus | Angular | ANG |
| 71, 72 | Precuneus | Precuneus | PCUN |
| 73, 74 | Paracentral lobule | Paracentral_Lobule | PCL |
| *75, 76* | *Caudate nucleus* | Caudate | CAU |
| *77, 78* | *Lenticular nucleus, Putamen* | Putamen | PUT |
| 79, 80 | Lenticular nucleus, Pallidum | Pallidum | PAL |
| 81, 82 | *Thalamus* | Thalamus | THA |
| 83, 84 | Heschl’s gyrus | Heschl | HES |
| 85, 86 | Superior temporal gyrus | Temporal_Sup | STG |
| 87, 88 | Temporal pole: superior temporal gyrus | Temporal_Pole_Sup | TPOsup |
| 89, 90 | Middle temporal gyrus | Temporal_Mid | MTG |
| 91, 92 | Temporal pole: middle temporal gyrus | Temporal_Pole_Mid | TPOmid |
| 93, 94 | Inferior temporal gyrus | Temporal_Inf | ITG |
| 95, 96 | Crus I of cerebellar hemisphere | Cerebellum_Crus1 | CERCRU1 |
| 97, 98 | Crus II of cerebellar hemisphere | Cerebellum_Crus2 | CERCRU2 |
| 99, 100 | Lobule III of cerebellar hemisphere | Cerebellum_3 | CER3 |
| 101, 102 | Lobule IV, V of cerebellar hemisphere | Cerebellum_4_5 | CER4_5 |
| 103, 104 | Lobule VI of cerebellar hemisphere | Cerebellum_6 | CER6 |
| 105, 106 | Lobule VIIB of cerebellar hemisphere | Cerebellum_7b | CER7b |
| 107, 108 | Lobule VIII of cerebellar hemisphere | Cerebellum_8 | CER8 |
| 109, 110 | Lobule IX of cerebellar hemisphere | Cerebellum_9 | CER9 |
| 111, 112 | Lobule X of cerebellar hemisphere | Cerebellum_10 | CER10 |
| 113 | Lobule I, II of vermis | Vermis_1_2 | VER1_2 |
| 114 | Lobule III of vermis | Vermis_3 | VER3 |
| 115 | Lobule IV, V of vermis | Vermis_4_5 | VER4_5 |
| 116 | Lobule VI of vermis | Vermis_6 | VER6 |
| 117 | Lobule VII of vermis | Vermis_7 | VER7 |
| 118 | Lobule VIII of vermis | Vermis_8 | VER8 |
| 119 | Lobule IX of vermis | Vermis_9 | VER9 |
| 120 | Lobule X of vermis | Vermis_10 | VER10 |

**Table S1b.** The extra anatomical regions defined in AAL3 in each hemisphere and their label ^17^. Column 4 provides a set of possible abbreviations for the anatomical descriptions. In AAL3, the label shown in column 3 is followed by the number shown in column 1. In most cases, the first number in a row is for the left hemisphere, and the second number is for the right hemisphere. This does not apply to the raphe nuclei, which are midline structures.

| NO. | ANATOMICAL DESCRIPTION | LABEL  AAL3.nii.gz | POSSIBLE  ABBREVIATION |
| --- | --- | --- | --- |
| 121, 122 | Thalamus, Anteroventral Nucleus | Thal_AV | tAV |
| 123, 124 | Lateral posterior | Thal_LP | tLP |
| 125, 126 | Ventral anterior | Thal_VA | tVA |
| 127, 128 | Ventral lateral | Thal_VL | tVL |
| 129, 130 | Ventral posterolateral | Thal_VPL | tVPL |
| 131, 132 | Intralaminar | Thal_IL | tIL |
| 133, 134 | Reuniens | Thal_Re | tRe |
| 135, 136 | Mediodorsal medial magnocellular | Thal_MDm | tMDm |
| 137, 138 | Mediodorsal lateral parvocellular | Thal_MDl | tMDl |
| 139, 140 | Lateral geniculate | Thal_LGN | tLGN |
| 141, 142 | Medial Geniculate | Thal_MGN | tMGN |
| 143, 144 | Pulvinar anterior | Thal_PuA | tPuA |
| 145, 146 | Pulvinar medial | Thal_PuM | tPuM |
| 147, 148 | Pulvinar lateral | Thal_PuL | tPuL |
| 149, 150 | Pulvinar inferior | Thal_PuI | tPuI |
| 151, 152 | Anterior cingulate cortex, subgenual | ACC_sub | ACCsub |
| 153, 154 | Anterior cingulate cortex, pregenual | ACC_pre | ACCpre |
| 155, 156 | Anterior cingulate cortex, supracallosal | ACC_sup | ACCsup |
| 157, 158 | Nucleus accumbens | N_Acc | Nacc |
| 159, 160 | Ventral tegmental area | VTA | VTA |
| 161, 162 | Substantia nigra, pars compacta | SN_pc | SNpc |
| 163, 164 | Substantia nigra, pars reticulata | SN_pr | SNpr |
| 165, 166 | Red nucleus | Red_N | RedN |
| 167, 168 | Locus coeruleus | LC | LC |
| 169 | Raphe nucleus, dorsal | Raphe_D | RapheD |
| 170 | Raphe nucleus, median | Raphe_M | RapheM |

**Table S2.** **Comparison of** **temporal variability between the group with chronic schizophrenia and the controls.**

| **Region** | **t value** | **p value** | **FDR p value** | **Temporal variability in controls** | **Temporal variability in patients** |
| --- | --- | --- | --- | --- | --- |
| Thal_VA | 3.58 | 0.00041 | 0.049 | 0.567 | 0.621 |
| Thal_VPL | 3.41 | 0.00075 | 0.049 | 0.556 | 0.599 |
| Thal_VA | 3.29 | 0.00116 | 0.049 | 0.569 | 0.619 |
| Thal_MDm | 3.17 | 0.00171 | 0.049 | 0.540 | 0.587 |
| OFCmed | 3.15 | 0.00185 | 0.049 | 0.651 | 0.693 |
| Thal_VL | 3.02 | 0.00275 | 0.055 | 0.524 | 0.574 |
| Thal_PuL | 3.01 | 0.00292 | 0.055 | 0.553 | 0.589 |
| Olfactory | 2.90 | 0.00412 | 0.065 | 0.647 | 0.685 |
| Thal_VL | 2.87 | 0.00440 | 0.065 | 0.524 | 0.567 |
| Thal_MDl | 2.83 | 0.00496 | 0.065 | 0.544 | 0.587 |

**Table S3.** **Comparison of temporal variability between the group with first episode schizophrenia and the controls.**

| **Region** | **t value** | **p value** | **FDR p value** | **Temporal variability in controls** | **Temporal variability in patients** |
| --- | --- | --- | --- | --- | --- |
| ACC_pre | -3.78 | 0.00020 | 0.016 | 0.687 | 0.646 |
| Frontal_Sup_Medial | -3.63 | 0.00034 | 0.016 | 0.680 | 0.639 |
| Frontal_Sup_Medial | -3.61 | 0.00037 | 0.016 | 0.685 | 0.646 |
| SupraMarginal | -3.36 | 0.00088 | 0.029 | 0.680 | 0.638 |
| ACC_sup | -2.99 | 0.00308 | 0.068 | 0.693 | 0.660 |
| ACC_pre | -2.98 | 0.00321 | 0.068 | 0.681 | 0.648 |
| Temporal_Pole_Sup | 2.94 | 0.00361 | 0.068 | 0.649 | 0.690 |
| Temporal_Pole_Sup | 2.81 | 0.00535 | 0.088 | 0.653 | 0.689 |
| ACC_sup | -2.76 | 0.00611 | 0.090 | 0.682 | 0.650 |
| Temporal_Sup | 2.60 | 0.00977 | 0.129 | 0.655 | 0.689 |

**Table S4.** **Comparison of temporal variability between the group with ADHD and the controls.**

| **Region** | **t value** | **p value** | **FDR p value** | **Temporal variability in controls** | **Temporal variability in patients** |
| --- | --- | --- | --- | --- | --- |
| SN_pc | -2.37 | 0.018 | 0.755 | 0.724 | 0.694 |
| Rectus | 2.34 | 0.020 | 0.755 | 0.638 | 0.680 |
| Cuneus | -2.13 | 0.034 | 0.755 | 0.625 | 0.609 |
| Cingulate_Post | 2.10 | 0.037 | 0.755 | 0.651 | 0.689 |
| Frontal_Med_Orb | 2.08 | 0.039 | 0.755 | 0.635 | 0.669 |
| Frontal_Med_Orb | 2.02 | 0.044 | 0.755 | 0.635 | 0.670 |
| Rectus | 2.02 | 0.044 | 0.755 | 0.652 | 0.683 |
| Lingual | -1.95 | 0.053 | 0.755 | 0.621 | 0.606 |
| Red_N | -1.92 | 0.056 | 0.755 | 0.709 | 0.682 |
| Calcarine | -1.91 | 0.057 | 0.755 | 0.648 | 0.630 |

**References**

1. Glasser, M.F.*, et al.* The minimal preprocessing pipelines for the Human Connectome Project. *Neuroimage* **80**, 105-124 (2013).

2. Cheng, W., Rolls, E.T., Ruan, H. & Feng, J. Functional connectivities in the brain that mediate the association between depressive problems and sleep quality. *JAMA Psychiatry* **75**, 1052-1061 (2018).

3. Rolls, E.T.*, et al.* Beyond the disconnectivity hypothesis of schizophrenia. *Cereb. Cortex* **30**, 1213-1233 (2020).

4. DSM-IV. *Diagnostic and Statistical Manual of Mental Disorders* (American Psychiatric Association, Washington, DC., 1984).

5. Kay, S.R., Fiszbein, A. & Opler, L.A. The positive and negative syndrome scale (PANSS) for schizophrenia. *Schizophr. Bull.* **13**, 261-276 (1987).

6. Du, J.*, et al.* The genetic determinants of language network dysconnectivity in drug-naïve early stage schizophrenia. *Schizophr. Bull.*, in review (2020).

7. DuPaul, G.J., Power, T.J., Anastopoulos, A.D. & Reid, R. *ADHD Rating Scale—IV: Checklists, norms, and clinical interpretation* (Guilford Press, 1998).

8. Cheng, W., Ji, X., Zhang, J. & Feng, J. Individual classification of ADHD patients by integrating multiscale neuroimaging markers and advanced pattern recognition techniques. *Front. Syst. Neurosci.* **6**, 58 (2012).

9. Navarro Schroder, T., Haak, K.V., Zaragoza Jimenez, N.I., Beckmann, C.F. & Doeller, C.F. Functional topography of the human entorhinal cortex. *Elife* **4** (2015).

10. Smith, S.M.*, et al.* Resting-state fMRI in the Human Connectome Project. *Neuroimage* **80**, 144-168 (2013).

11. Salimi-Khorshidi, G.*, et al.* Automatic denoising of functional MRI data: combining independent component analysis and hierarchical fusion of classifiers. *Neuroimage* **90**, 449-468 (2014).

12. Griffanti, L.*, et al.* ICA-based artefact removal and accelerated fMRI acquisition for improved resting state network imaging. *Neuroimage* **95**, 232-247 (2014).

13. Colclough, G.L.*, et al.* The heritability of multi-modal connectivity in human brain activity. *Elife* **6** (2017).

14. Vidaurre, D.*, et al.* Discovering dynamic brain networks from big data in rest and task. *Neuroimage* **180**, 646-656 (2018).

15. Smith, S.M.*, et al.* A positive-negative mode of population covariation links brain connectivity, demographics and behavior. *Nat. Neurosci.* **18**, 1565-1567 (2015).

16. Rolls, E.T., Joliot, M. & Tzourio-Mazoyer, N. Implementation of a new parcellation of the orbitofrontal cortex in the automated anatomical labeling atlas. *Neuroimage* **122**, 1-5 (2015).

17. Rolls, E.T., Huang, C.C., Lin, C.P., Feng, J. & Joliot, M. Automated anatomical labelling atlas 3. *Neuroimage* **206**, 116189 (2020).
